# Clinical Characteristics and Disease Burden of Wheat Allergy Dependent on Augmentation Factors in Recreationally Active and Trained Individuals

**DOI:** 10.1111/sms.70134

**Published:** 2025-09-10

**Authors:** Valentina Faihs, Claudia Kugler, Rebekka K. Linhart, Julia Felicitas Pilz, Tilo Biedermann, Knut Brockow

**Affiliations:** ^1^ Department of Dermatology and Allergy Biederstein, School of Medicine and Health TUM University Hospital Rechts der Isar Munich Germany; ^2^ Department of Dermatology and Allergy Centre, Odense Research Center for Anaphylaxis (ORCA) Odense University Hospital Odense Denmark

**Keywords:** allergy, anaphylaxis, athletes, exercise, quality of life, WALDA, WDEIA, wheat allergy

## Abstract

In wheat allergy dependent on augmentation factors (WALDA), allergic reactions occur when wheat ingestion is combined with exercise or rarely other augmentation factors. We analyzed clinical characteristics and disease burden in recreationally active and trained individuals with WALDA diagnosed by oral challenge test. Clinical characteristics, serological data, and quality of life (QOL) questionnaires were analyzed and completed with follow‐up interviews. Twenty recreationally active and trained WALDA patients (five female, 15 male; median age 45 years; median exercise frequency 3.5 times weekly) participated. All had experienced allergic reactions during or after different types of exercise—predominantly endurance activities—and 85% developed systemic anaphylaxis. Diagnosis was delayed by a median of 5 years, during which 35% reduced or discontinued exercise out of fear of further reactions. QOL significantly decreased after initial reactions (*p* < 0.001). In 95%, symptoms began during exercise, primarily with endurance activities. Interestingly, 40% identified weight training as never triggering reactions. In the diagnostic challenge tests, 90% reacted to wheat gluten alone in high doses or in combination with acetylsalicylic acid. Following diagnosis, QOL significantly improved (*p* < 0.001), and fear of reactions decreased (*p* = 0.01). During follow‐up (median 18 months), all were able to resume exercise with dietary modifications alone; 40% remained reaction‐free, while others experienced only mild urticaria during accidental reactions. Thus, WALDA significantly impacts recreationally active and trained individuals both physically and psychologically. Weight training may be less likely to trigger reactions than endurance activities. Timely diagnosis through challenge tests and comprehensive education and management effectively restore exercise participation and QOL.

## 1 Introduction

Anaphylaxis is a potentially fatal allergic reaction with an acute onset of symptoms involving different organ systems and requiring immediate medical treatment [1]. Beyond its immediate life‐threatening nature, anaphylaxis is often accompanied by a significant decrease in overall quality of life (QOL) and the fear of further reactions [2, 3, 4]. This may apply in a special way to recreationally active and trained individuals who experience allergic reactions in the context of their exercise routine [5].

**FIGURE 1:**
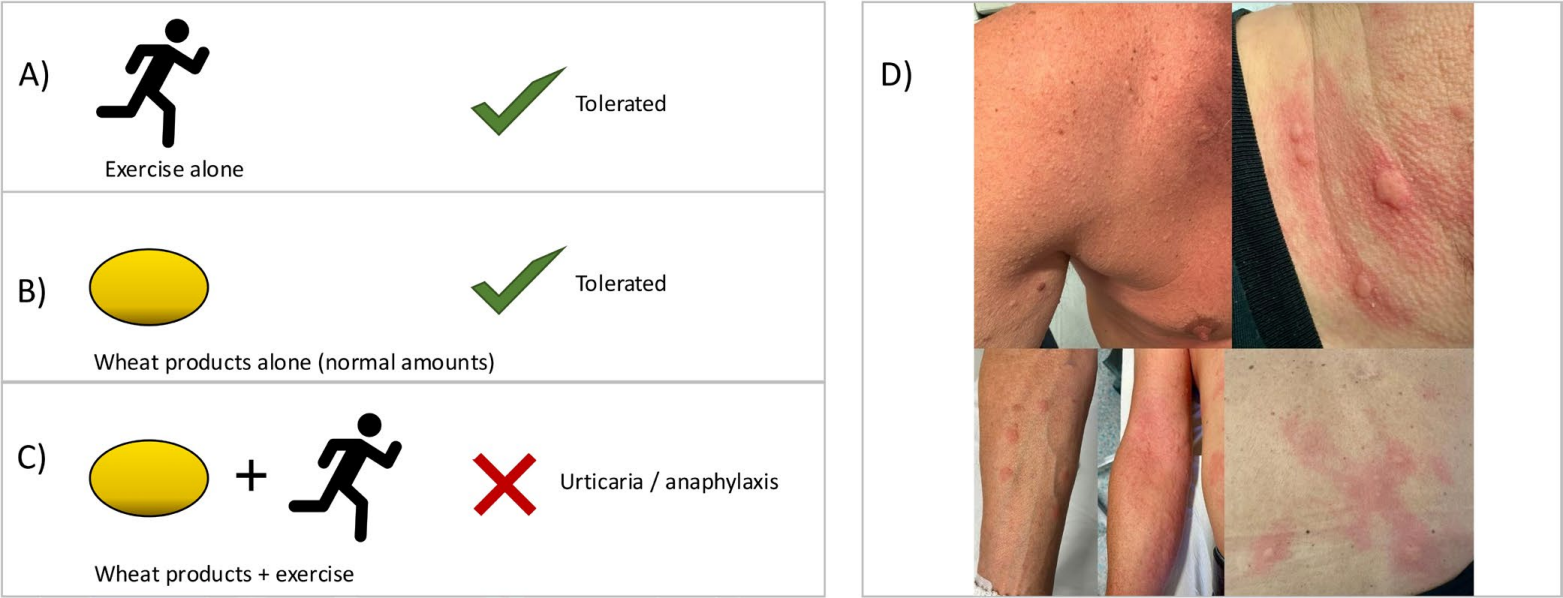
Overview of the clinical presentation in wheat allergy dependent on augmentation factors (WALDA). (A) Exercise alone is well tolerated by patients. (B) Normal amounts of wheat products are tolerated when consumed without augmentation factors. (C) The combination of wheat products and exercise triggers allergic reactions, ranging from urticaria to life‐threatening anaphylaxis. (D) Clinical pictures of urticaria of WALDA patients in oral challenge tests.

Wheat is one of the most common elicitors of food‐induced anaphylaxis in adults [6]. In adults, wheat allergy predominantly presents as wheat allergy dependent on augmentation factors (WALDA) [7]. In this IgE‐mediated wheat allergy, allergic reactions occur only when wheat is combined with augmentation factors like exercise, while wheat products alone are typically tolerated [7] (Figure 1A–C). The symptom severity may range from urticaria or angioedema alone (Figure 1D) to severe, life‐threatening reactions with cardiovascular and respiratory involvement [7, 8, 9].

Exercise represents the most frequently reported augmentation factor, leading to the frequently used description of the disease as wheat‐dependent exercise‐induced anaphylaxis (WDEIA) [7, 10]. However, other potential augmentation factors exist, such as nonsteroidal anti‐inflammatory drugs (NSAID), alcohol, or infections [11, 12]. While the precise pathophysiological mechanism behind augmentation factors remains elusive, one primary hypothesis suggests an enhanced gastrointestinal allergen absorption [13]. Additionally, some patients with only skin‐related symptoms (urticaria/angioedema) may not fulfill the anaphylaxis criteria in all or some episodes. Thus, WALDA represents the more accurate terminology when describing the disease [14].

The first steps to a correct diagnosis involve taking an accurate medical history and assessing the patient's sensitization profile. Specific IgE (sIgE) against ω5‐gliadin—the major allergen in wheat gluten—is often used as a screening parameter for WALDA, showing a high sensitivity and specificity [13]. In an oral challenge test (OCT) with wheat gluten alone and stepwise addition of augmentation factors, the diagnosis can be confirmed and the patients' individual reaction threshold can be detected [13, 15, 16, 17].

The disease typically develops during adulthood and is often diagnosed with a substantial delay [4, 10]. In the meantime, the threat of recurrent reactions often severely impairs the patients' daily life [4, 18, 19, 20]. As exercise represents the main eliciting augmentation factor in WALDA, affected recreationally active and trained individuals may suffer from a particularly high disease burden. It remains unclear whether different types of exercise have the same potential to elicit reactions.

Despite its clinical significance, WALDA has not yet been investigated in the specific context of recreationally active and trained individuals. Therefore, we aimed to investigate the clinical characteristics and disease burden of active individuals suffering from WALDA.

## 2 Methods

### 2.1 Study Population

Between January 2019 and April 2024, recreationally active and trained individuals with challenge‐confirmed WALDA were included in this study at the Department of Dermatology and Allergy Biederstein of the Technical University of Munich, Germany. Patients were recruited consecutively from our allergy department, including self‐referred patients, those presenting to the outpatient clinic of our institution, and referrals from primary care physicians and specialists.

The following inclusion criteria were applied: (1) a history of allergic reactions after consuming wheat in combination with augmentation factors despite regular tolerance of wheat products alone, (2) sIgE levels against ω5‐gliadin ≥ 0.35 kU/L (ImmunoCAP assay, Thermo Fisher Scientific, Uppsala, Sweden), (3) a positive OCT to wheat gluten alone or in combination with augmentation factors, (4) a regular, weekly exercise routine with ≥ 2 sessions per week, and (5) written informed consent to participate in the study. The study received approval from the local medical ethics committee (approval number 477/21S‐NP).

### 2.2 Clinical History, Laboratory Allergy Diagnostics, and Skin Testing

A comprehensive medical history was taken from all patients through structured interviews at initial presentation, including self‐reported information on the severity of the most severe reaction according to Ring and Messmer [21], the augmentation factors eliciting reactions, the type of exercise regularly performed, the types and intensity of activities eliciting reactions, and the timespan between the first allergic reaction and the final diagnosis. Participants were classified according to the McKay et al. Participant Classification Framework [22] based on training frequency, sport identification, and competition involvement. Blood samples were collected to determine total IgE and sIgE levels against wheat, ω5‐gliadin, and timothy grass, as well as basal serum tryptase levels (ImmunoCAP assay, Thermo Fisher Scientific, Uppsala, Sweden). Prick‐to‐prick skin tests (PPST) were performed with wheat gluten as well as with histamine dihydrochloride (10 mg/mL; ALK‐Abello, Copenhagen, Denmark) as a positive control and 0.9% saline as a negative control. Results with a mean wheal diameter of 3 mm or greater compared to the negative control after 20 min were considered positive [23]. After providing informed consent to participate in the research study, patients were followed up with phone interviews.

### 2.3 Oral Challenge Tests (OCT)

The OCT were performed in an inpatient setting following a previously published protocol [13]. In short, gluten buns were prepared by mixing wheat gluten with double the amount of water and successive baking. The OCT followed a stepwise approach with increasing allergen doses and, if tolerated, sequential addition of augmentation factors. First, the patients were challenged with wheat gluten alone in increased doses, reaching allergen amounts exceeding those consumed in a regular diet, with 8, 16, and 32 g of wheat gluten given at time intervals of at least 60 min. In case of tolerance, on the next day, 1000 mg of acetylsalicylic acid (ASA) was added as an augmentation factor, followed by increasing doses of wheat gluten (8 g, 16 g, and 32 g). Intolerance to ASA was ruled out either by a history of regularly tolerated NSAID or by separate OCT with ASA alone. In case of no reaction, the OCT was continued with sequential addition of different augmentation factors: 1000 mg of ASA and 20 mL of 95% ethanol (Braun, Melsungen, Germany) in fruit or peppermint infusion were administered, followed by 32 g of wheat gluten after 30 min. In case of tolerance, 20 min of exercise on a treadmill was added after 60 min. This last challenge step may be repeated with 64 g of wheat gluten. Patients were motivated to exercise with at least moderate to high subjective intensity, with a perceived level of exertion of at least 13 points on the Borg scale [24]. In the case of an objective allergic reaction, the OCT was immediately stopped, and antiallergic treatment was performed. Reaction thresholds were recorded on an ordinal scale ranging from 1 to 8, according to [4].

### 2.4 Quality of Life (QOL) Questionnaires

The Food Allergy Quality of Life Questionnaire‐Adult Form (FAQLQ‐AF) and the WALDA post‐challenge questionnaire were used to assess the patients' QOL and their fears and perceptions of the OCT after the diagnosis of WALDA was confirmed.

The FAQLQ‐AF is an internationally used questionnaire to measure health‐related QOL in food allergy [25]. In this study, the validated German version was used [26]. Twenty‐nine items must be answered on a response scale ranging from 1 to 7, with higher values indicating an increasing effect on the health‐related QOL. The FAQLQ‐AF can be subdivided into four domains: allergen avoidance and dietary restrictions (AADR, 11 items), risk of accidental exposure (RAE, eight items), food allergy‐related health (FAH, three items), and emotional impact (EI, seven items). According to the literature, the total FAQLQ‐AF score and the score of each domain were calculated as the average score of the individual items.

Additionally, a post‐challenge questionnaire developed especially for WALDA patients was used to assess the patients' fears and overall QOL, as well as the burden and benefit of the challenge‐confirmed diagnosis, as described in [4] and in Table S2.

### 2.5 Statistics

SPSS Statistics 29.0 (SPSS Inc., Chicago, USA) and GraphPad PRISM version 10 (GraphPad Software Inc., La Jolla, CA, USA) were used for statistical analyses. Patient characteristics are presented as median with range for continuous variables and as frequencies with percentages for categorical variables. For the FAQLQ‐AF, both median with interquartile range (IQR) and mean with standard deviation (SD) are reported for total and domain scores. Differences between paired measurements were analyzed using Wilcoxon signed‐rank tests. Correlations were assessed using Spearman's rank correlation coefficients. A two‐sided *p*‐value < 0.05 was considered statistically significant.

## 3 Results

### 3.1 Study Population

Twenty adult recreationally active and trained individuals (five female, 15 male; median age 45 years, range 23–62) with challenge‐confirmed WALDA were included in the study. During the study period, 19 patients with challenge‐confirmed WALDA were not included due to too low exercise frequency (< 2×/week; inclusion criterion 4) while meeting all other inclusion criteria. No patients were excluded after meeting inclusion criteria, and there were no dropouts during the study period. Participants reported exercising 3.5 times weekly (range 2–7 sessions) by engaging in various physical activities, as detailed in Table S1. According to the McKay et al. classification framework [22], 15 patients (75%) were classified as Tier 1 (“Recreationally Active”) and 5 (25%) as Tier 2 (“Trained/Developmental”) (Table S1).

### 3.2 Clinical Characteristics and Allergological Work‐Up

Table 1 gives an overview of the clinical characteristics of the included patients. According to the study inclusion criteria, all patients showed elevated sIgE levels against ω5‐gliadin (≥ 0.35 kU/L) and objective allergic reactions in the OCT (20/20 patients). Only 12/20 patients (60%) showed elevated sIgE against wheat (≥ 0.35 kU/L). Nine patients self‐reported atopic comorbidities (Table 1), of which 8 (40% of included patients) reported hay fever symptoms. Out of all included patients, 12 (60%) showed elevated sIgE against timothy grass (≥ 0.35 kU/L). However, timothy grass sIgE levels showed no correlation with either reaction frequency before diagnosis or reaction severity (*r* = −0.06 and −0.17, respectively; n.s.). Basal serum tryptase levels significantly correlated with the level of the most severe reaction in the patients' histories according to Ring and Messmer (*r* = 0.532, *p* = 0.016; all tryptase values below the threshold of 11.4 mg/L). A nonsignificant trend was found between higher sIgE levels against ω5‐gliadin and higher reaction severity in the patients' histories (*r* = 0.421, *p* = 0.06).

**TABLE 1:** Clinical characteristics of recreationally active and trained individuals suffering from WALDA.

Variable	WALDA patients (*n* = 20)
Age, median (range)	45 years (23–62)
Sex, male/female, *n* (%)	15/5 (75%/25%)
Any self‐reported atopic comorbidities^a^, *n* (%)^c^	9 (45%)
Exercise sessions per week, median (range)^c^	3.5 (2–7)
Years to diagnosis, median (range)^d^	5 (0.5–24)
Severity of most severe reaction^b^, *n* (%)^c^	Grade I: 3 (15%) Grade II: 5 (25%) Grade III: 11 (55%) Grade IV: 1 (5%)
Self‐reported number of allergic reactions, until diagnosis, median (range)^c^	10 reactions (2–60)
Total IgE, median (range)	258 kU/L (52–2848)
sIgE against wheat, median (range)	0.6 kU/L (< 0.1–5.3)
sIgE against ω5‐gliadin, median (range)	5.1 kU/L (0.5–32.2)
Positive PPST with wheat gluten, *n* (%)	18 (90%)
Basal serum tryptase, median (range)	3.8 μg/L (2.1–8.5)
Positive OCT, *n* (%)	20 (100%)
Reaction threshold, *n* (%)	(1) 8 g wheat gluten: 2 (10%) (2) 16 g wheat gluten: 2 (10%) (3) 32 g wheat gluten: 4 (20%) (4) ASA + 8 g wheat gluten: 7 (35%) (5) ASA + 16 g wheat gluten: 3 (15%) (6) ASA + 32 g wheat gluten: 0 (0%) (7) ASA + alcohol + 32 g wheat gluten + exercise: 2 (10%) (8) ASA + alcohol + 64 g wheat gluten + exercise: 0 (0%)

Abbreviations: ASA, acetylic salicylic acid; OCT, oral challenge test; PPST, prick‐to‐prick skin test; WALDA, wheat allergy dependent on augmentation factors.

^a^ Defined as the presence of hay fever, allergic bronchial asthma, and/or atopic eczema.

^b^ According to the Ring and Messmer scale.

^c^ Data self‐reported by patients during structured interviews.

^d^ Calculated from first self‐reported reaction to challenge‐confirmed diagnosis.

In the diagnostic OCT, in 40% of patients (*n* = 8), reactions were achieved already after ingesting pure wheat gluten due to the higher allergen content than that commonly consumed with commercial, unconcentrated wheat products. Adding ASA as an augmentation factor could confirm the diagnosis in a further 50% of the patients (*n* = 10), while additional exercise and alcohol were needed in two patients (10%). 19/20 patients reacted with only urticaria, while one patient developed additional abdominal pain and hypotension, which rapidly subsided after antiallergic treatment.

A median of 5 years (range 0.5–24) passed between the first allergic reaction and the correct diagnosis, with 30% of patients (*n* = 6) being diagnosed with a delay of 17 years or more (Table 1). This led to a median of 10 allergic reactions per patient until the diagnosis of WALDA.

### 3.3 Augmentation Factors in the Patients´ Histories

**FIGURE 2:**
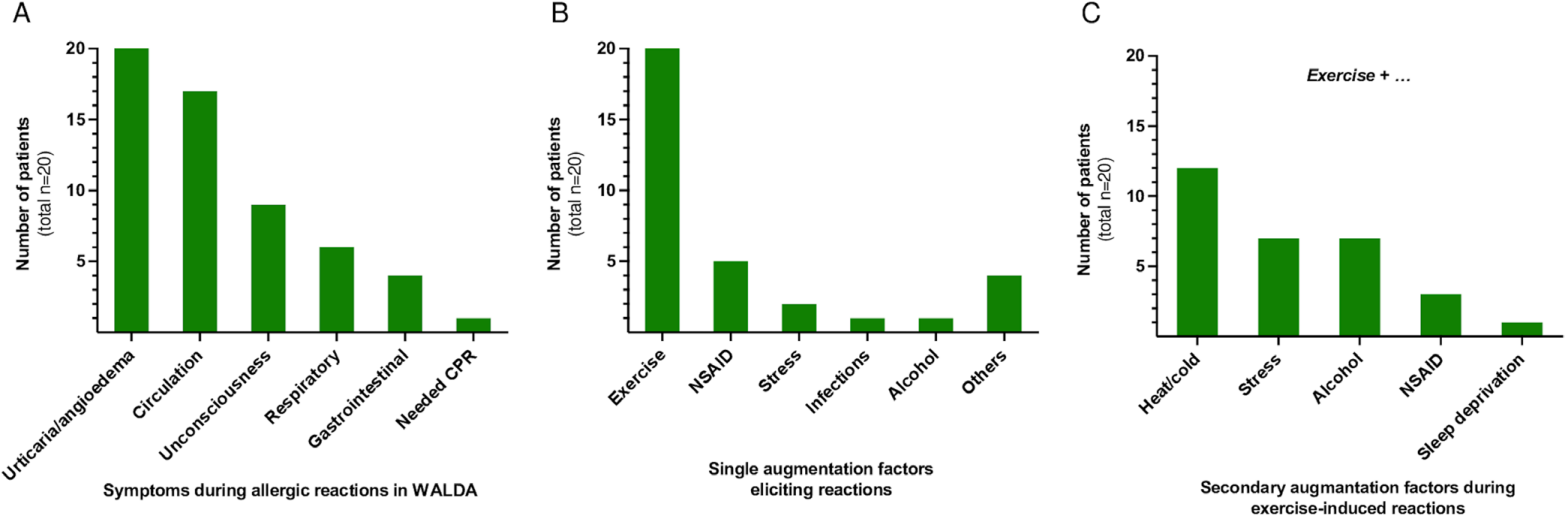
Clinical symptoms and augmentation factors reported from recreationally active and trained individuals suffering from WALDA. (A) Symptoms experienced during allergic reactions due to WALDA. (B) Single augmentation factors that triggered allergic reactions in patients' histories. (C) Secondary augmentation factors in addition to exercise involved in reactions in the patients' histories.

All patients had suffered from allergic reactions when consuming wheat in combination with augmentation factors despite regular tolerance of wheat products alone. The median reported timespan between wheat consumption and symptom onset was 60 min (range 10–300 min). All patients (20/20) had developed urticaria during their previous reactions; however, it remained the only symptom in just 15% (*n* = 3). 85% of patients (*n* = 17) had indeed experienced systemic anaphylaxis (Table 1, Figure 2A). One patient even suffered from three episodes of Grade 4 anaphylaxis with the need for cardiopulmonary resuscitation.

All included patients had experienced allergic reactions during exercise (Figure 2B). 45% of patients (*n* = 9) reported allergic reactions outside the context of exercise, with different augmentation factors involved (Table S1, Figure 2B). The most frequently reported single augmentation factor after exercise was NSAID, which led to reactions in 25% of patients (5/20), followed by psychological stress (in 2/20 as single augmentation factor, in one additional case in combination with NSAID and infection). In addition, reactions were reported after wheat consumption in combination with alcohol alone, tolperisone (muscle relaxant), sauna, infections, and a vaccination, each reported once.

However, even if reactions had happened in the context of exercise, most patients (17/20, 85%) identified potential secondary augmentation factors at least in some of their prior reactions: temperature extremes (heat/cold) were named most frequently, followed by psychological stress, alcohol, NSAID, and sleep deprivation, as shown in Figure 2C. Patient histories revealed reactions occurring across all seasons and during outdoor as well as indoor activities.

### 3.4 Exercise‐Related Findings in Recreationally Active and Trained Individuals Suffering From WALDA

All recreationally active and trained individuals with challenge‐confirmed WALDA experienced allergic reactions when consuming wheat before exercise. For 55% (11/20) of participants, exercise was the only identified augmentation factor (Table S1).

Despite engaging in a variety of physical activities, patients mainly experienced allergic reactions after wheat consumption in combination with endurance‐type exercises such as running, cycling, walking, hiking, ski touring, soccer, and swimming (Table S1). When asked about types of exercise that had been identified never to elicit reactions, 40% of patients (8/20) named weight training. Even more, none of the study participants reported any allergic reactions occurring in the context of weight training sessions.

In 95% of patients (19/20), symptoms consistently began during exercise. Symptoms occurred across a range of exercise intensities, with individual patients experiencing reactions at different intensity levels (low to high, often varying between episodes). The time between starting exercise and symptom onset varied widely among patients (10–120 min, median 30 min). Only one patient (5%) experienced a distinct pattern where all allergic reactions (approximately 10 episodes) consistently occurred approximately 5 to 15 min after completing their jogging activity. Interestingly, this unique postexercise reaction pattern was confirmed during the patient's OCT, when symptoms appeared 4 min after completing 20 min of intensive treadmill running that followed administration of 1000 mg ASA, 20 mL alcohol, and 32 g wheat gluten.

### 3.5 Quality of Life (QOL) and Psychological Impact

The responses given in the WALDA post‐challenge questionnaire are summarized in Table S2.

**FIGURE 3:**
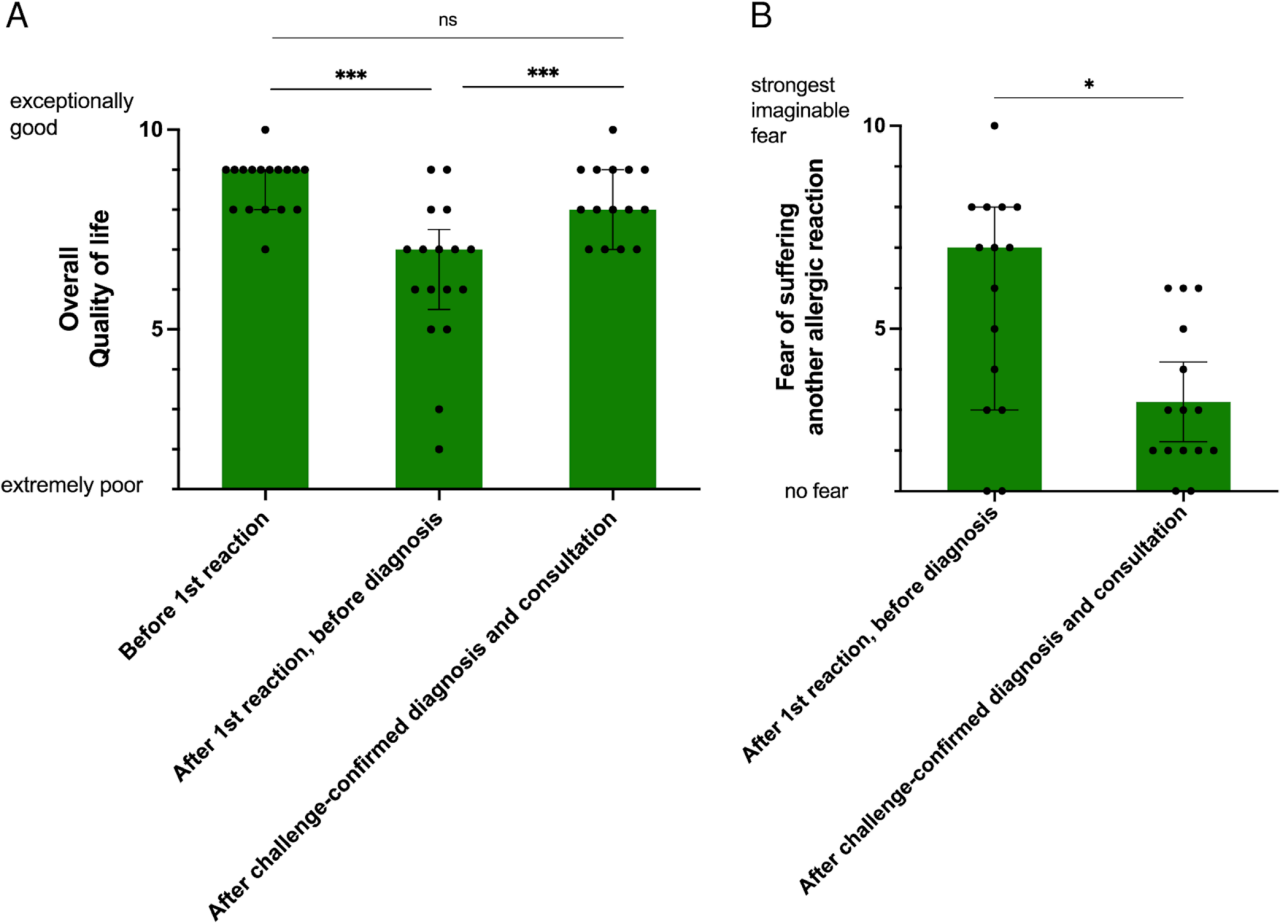
(A) Self‐reported overall quality of life in recreationally active and trained individuals suffering from WALDA before their first allergic reaction, after the first reaction, and after the diagnosis by oral challenge test and consultation. (B) Self‐reported fear of experiencing further allergic reactions in recreationally active and trained individuals suffering from WALDA before and after challenge‐confirmed diagnosis. *p < 0.05, ***p < 0.001, compared with previous time point. Data are shown as individual values and median with interquartile range. Missing values: 2–4.

After experiencing their first allergic reaction, patients reported a severe decrease in their overall QOL (*p* < 0.001; Figure 3A). However, following correct diagnosis through OCT and education about their disease, patients experienced significant recovery of their previous QOL (*p* < 0.001 compared to pre‐diagnosis levels; Figure 3A).

Table 2 presents the FAQLQ‐AF scores (total and domains) of active individuals with WALDA after diagnosis.

**TABLE 2:** FAQLQ‐AF scores of challenge‐confirmed WALDA patients (total and domains).

	FAQLQ‐AF total score	AADR	RAE	FAH	EI
Median (IQR)	3.69 (1.38)	3.82 (2.19)	4.25 (1.75)	4.00 (2.34)	3.43 (1.28)
Mean (SD)	3.83 (1.06)	3.78 (1.18)	3.99 (1.28)	4.13 (1.36)	3.60 (1.26)

Abbreviations: AADR, allergen avoidance and dietary restrictions; EI, emotional impact; FAH, food allergy‐related health; FAQLQ‐AF, food allergy quality of life questionnaire—adult form; IQR, interquartile range; RAE, risk of accidental exposure; SD, standard deviation.

The first allergic reaction with an unknown trigger generated intense fear of recurrence in most patients (Figure 3B). This fear prompted six patients (30%) to reduce their exercise routine, while one patient (5%) completely discontinued all physical activity until receiving a confirmed diagnosis (Table S1). After challenge‐confirmed diagnosis and comprehensive education about WALDA, patients reported significantly reduced fear of future reactions (*p* = 0.01; Figure 3B).

Patients perceived the OCT procedure very positively, rating its benefit at the maximum median score of 10 out of 10 (range 5–10) while reporting minimal stress during testing (median 2 points, range 1–5).

### 3.6 Management and Follow‐Up of Patients

Following the confirmation of the diagnosis of WALDA, all patients were prescribed an emergency set for self‐treatment containing an adrenaline autoinjector with adequate instruction and were educated about the disease, including individual dietary counseling. In the post‐challenge questionnaire, most patients reported feeling well or very well informed about WALDA and current research (median 9 out of 10, range 4–10) and confident in dealing with their illness (median 9, range 6–10) (Table S2).

Patients were advised to reduce gluten‐containing products to individually tolerated amounts based on their reaction thresholds and to separate consumption from augmentation factors such as exercise by at least 4 to 6 h [13, 17]. However, one patient (Patient 1) chose a completely gluten‐free diet due to severe anxiety about future reactions. All patients successfully resumed their exercise routines with only dietary modifications.

No severe reactions occurred after a median follow‐up of 18 months. 40% of patients (*n* = 8) remained completely reaction‐free, while the rest experienced single episodes with mild urticaria during accidental reactions (e.g., wheat intake followed by unplanned exercise).

## 4 Discussion

This first comprehensive study of WALDA in recreationally active and trained individuals demonstrates that the disease leads to severe anaphylaxis in most affected patients, significantly decreases QOL, and creates substantial diagnostic challenges with years of delay before proper diagnosis. Additionally, this study reveals novel findings about triggering exercise modalities and effective management strategies to restore the QOL and exercise participation of active WALDA patients.

WALDA leads to severe and potentially life‐threatening reactions when wheat is consumed in combination with augmentation factors like exercise. Wheat represents one of the primary elicitors of food‐induced anaphylaxis in Europe [6], with reactions more severe than those elicited by other triggers [8]. This may be particularly relevant among recreationally active and trained individuals, where allergic diseases already represent a relevant burden [27]. Previous research among Olympic athletes identified food allergy and anaphylaxis prevalence rates of 7.1% and 1.1%, respectively [28].

All patients in our cohort reported exercise as an augmentation factor, which aligns with previous literature identifying exercise as the predominant trigger across unselected WALDA populations [8, 9, 29]. Consistent with Kulhanan et al. [17], the reactions in the patients' histories were triggered by diverse sports activities, primarily endurance‐based, across various intensity levels.

Notably, weight training was frequently identified as an exercise type that never triggered reactions in patients' histories. To our knowledge, this is the first documentation suggesting weight training may pose a lower risk to induce allergic reactions in WALDA patients. While one WALDA case report mentions “exercise at the gymnasium” without further clarification [30], to our knowledge, no further documentation of weight training acting as an augmentation factor appears in the literature. This finding warrants further investigation through standardized OCT to determine whether the resistance focused, intermittent nature of weight training represents a lower‐risk activity for WALDA patients.

Several hypotheses regarding augmentation factor mechanisms have been proposed. One main hypothesis suggests that they may enhance allergen absorption across the gut barrier through increased gastrointestinal permeability, allowing food allergens to reach threshold levels necessary for triggering reactions [17, 31, 32]. Alternative proposed mechanisms include exercise‐induced blood flow redistribution, changes in plasma osmolality, and exercise‐induced acidosis [17, 31]. Building on these mechanisms, the differential triggering potential between endurance exercise and weight training observed in this study could be explained by endurance exercise potentially causing more sustained splanchnic hypoperfusion, increasing intestinal permeability [33], more robust hemodynamic redistribution enhancing allergen delivery to effector sites, and stronger cellular immune mobilization compared to the intermittent nature of resistance training [34]. Elucidating the precise mechanisms of augmentation factors would not only improve our understanding of the disease but could also help assess whether in WALDA, weight training brings a lower risk compared to endurance activities after wheat consumption.

Beyond exercise, nearly half of patients (45%) reported reactions triggered by other augmentation factors, with NSAID emerging as the second most common trigger. This aligns with Christensen et al.'s [12] finding that ASA is a potent augmentation factor in WALDA, potentially equaling or exceeding exercise in eliciting reactions. Most patients reported multiple augmentation factors working in combination, even in exercise‐related reactions. This synergistic effect was previously documented by Christensen et al. [12] who demonstrated increased reaction likelihood when combining exercise with ASA in OCT. The high prevalence of NSAID use among athletes—reported at 92% during competitive seasons even among youth athletes [35]—thus creates a particularly high‐risk profile for this population.

In line with research in general WALDA populations, this study reveals significant QOL deterioration [4, 19, 20] and high levels of fear [4] among recreationally active and trained individuals following initial reactions. This fear led to significant behavioral modifications, with 30% of active individuals reducing their exercise regimens and 5% abandoning physical activity entirely before correct diagnosis. Consistent with previous reports [10, 15, 29], this study also found a significant diagnostic delay of many years, with WALDA patients often being misdiagnosed with idiopathic anaphylaxis [36], NSAID hypersensitivity [36], recurrent acute urticaria [9], or non‐food‐dependent exercise‐induced anaphylaxis. Until diagnosis, recreationally active and trained individuals face the unique burden of choosing between maintaining fitness and avoiding potentially life‐threatening reactions.

Our findings demonstrate that in recreationally active and trained WALDA patients, challenge‐confirmed diagnosis and comprehensive patient education not only provide diagnostic certainty but also significantly improve patients' QOL and reduce their fear of further reactions. Although the end point of OCT is an objective reaction, recreationally active and trained individuals rated the diagnosis by OCT as highly beneficial, as described before in general WALDA populations [4]. Especially for active individuals, the OCT provides crucial reassurance to enable them to return to exercise routines with appropriate precautions, replacing activity avoidance driven by diagnostic uncertainty and fear.

Notably, despite exercise being the primary augmentation factor in patients' histories, the diagnostic OCT revealed that in 90% of patients, reactions could be elicited with high doses of wheat gluten alone or combined with ASA, with only two cases requiring additional exercise. Previous research has demonstrated that in WALDA, reactions can be triggered even without augmentation factors by substantially increasing the allergen dose, which can be achieved by using pure wheat gluten instead of wheat products [13, 37]. Due to the interchangeable potential of different augmentation factors to elicit reactions [12], ASA has been integrated as the first augmentation factor in the OCT protocol as it is easier to administer than exercise in daily clinical practice [13].

Following diagnosis, WALDA patients require comprehensive management, including emergency medication, detailed education about their condition, and personalized dietary counseling based on their OCT‐determined reaction threshold [13, 38]. Dietary management typically follows one of two approaches. Most patients are advised to adopt a situational avoidance strategy, limiting gluten‐containing cereals to individually tolerated amounts and separating wheat consumption from augmentation factors (such as exercise) by 4 to 6 h [13, 17]. Research suggests this approach may maintain or increase the patient's reaction threshold over time [13, 39]. Thus, this was the approach used for all but one patient in this study. Alternatively, patients with very low reaction thresholds or those preferring it for psychological reasons (like one patient in this study) may choose a completely gluten‐free diet. Literature indicates accidental reactions occur with both approaches in daily life, whether from unexpected augmentation factors or inadvertent wheat consumption [10, 40].

In this study's patient cohort follow‐up, the management approach proved effective. Only mild urticaria was reported during accidental exposures, which patients could effectively treat themselves, and 40% remained completely reaction‐free. Notably, all active individuals successfully resumed their exercise routines while following dietary modifications, demonstrating that proper diagnosis and management can effectively restore regular physical activity without compromising safety.

Several limitations must be acknowledged. The monocentric design and relatively small sample size necessitate a cautious interpretation of our findings. However, these limitations are inherent to studying WALDA, which remains underdiagnosed with small patient cohorts in most centers—particularly when focusing on recreationally active and trained individuals with challenge‐confirmed diagnosis. Most significantly, the observed differences between endurance activities and weight training were based on patient reports rather than standardized challenge testing. Additionally, the retrospective assessment of QOL changes may be subject to recall bias given the substantial diagnostic delays.

Despite these limitations, our findings provide valuable insights into the specific challenges recreationally active and trained WALDA patients face, with important implications for diagnosis, management, and QOL. Increased awareness of this condition among healthcare providers is crucial for the proper identification and management of affected patients.

## 5 Perspective

WALDA significantly impacts recreationally active and trained individuals, causing anaphylaxis in most cases and substantially reducing QOL. Diagnosis is typically delayed by many years, resulting in numerous allergic episodes and forcing many active individuals to limit or abandon exercise until being correctly diagnosed.

Even though exercise was the primary augmentation factor in patients' histories, in most patients, the diagnosis can be confirmed without exercise in OCT using wheat gluten alone or combined with ASA, reflecting the interchangeable nature of augmentation factors.

The novel finding that weight training may be less likely to trigger reactions than endurance activities warrants further investigation to identify safer exercise modalities and understand underlying mechanisms.

Following challenge‐confirmed diagnosis and comprehensive education, patients experienced significant improvement in QOL and reduced fear of further reactions. With proper dietary counseling tailored to individual reaction thresholds, all patients successfully resumed their exercise routines without severe allergic reactions.

These findings emphasize the importance of awareness among healthcare professionals. Early recognition, challenge‐confirmed diagnosis, and comprehensive management represent the optimal pathway for recreationally active and trained individuals with WALDA to avoid life‐threatening reactions, restore their QOL, and enable them to resume their former exercise routine.

## 6 Conclusion

WALDA significantly impacts recreationally active and trained individuals, with most experiencing systemic anaphylaxis and substantial diagnostic delays. Weight training appears less likely to trigger reactions than endurance activities. Challenge‐confirmed diagnosis and individualized dietary management effectively restore exercise participation and QOL. Early recognition and proper management enable WALDA patients to safely maintain active lifestyles through dietary modifications rather than activity restriction.

## Author Contributions

V.F. carried out the investigation and OCT performed the conceptualization, conducted data curation and visualization, executed formal analysis, and drafted the original manuscript; C.K. contributed to conceptualization, performed dietary counseling, and reviewed and edited the manuscript; R.K.L. performed formal analysis and participated in reviewing and editing the manuscript; J.F.P. conducted formal analysis and contributed to reviewing and editing the manuscript; T.B. participated in conceptualization, acquired funding, reviewed and edited the manuscript, and provided supervision; K.B. contributed to conceptualization, acquired funding, developed methodology, supervised the work, and drafted the original manuscript. All authors have read and approved the final version of the manuscript and agree with the order of presentation of the authors.

## Ethics Statement

The study was approved by the Ethics Committee of the Technical University of Munich (approval number 477/21 S‐NP).

## Consent

Informed consent was obtained from all patients involved in the study.

## Conflicts of Interest

All authors declare no conflicts of interest.

## Supporting information


**Table S1:** Overview of the augmentation factors and clinical histories of recreationally active and trained individuals with challenge‐confirmed WALDA.
**Table S2:** Patient‐reported outcomes from recreationally active and trained individuals with WALDA: Responses to post‐challenge questionnaire (as described in ^2^).

## Data Availability

The data that support the findings of this study are available from the corresponding author upon reasonable request.
