# Supplementary material for: Clinical Characteristics and Disease Burden of Wheat Allergy Dependent on Augmentation Factors in Recreationally Active and Trained Individuals
Source: Scand J Med Sci Sports. 2025 Sep 10;35(9):e70134. doi: 10.1111/sms.70134 (PMC12421479; doi:10.1111/sms.70134)
Supplement: Supplementary file 1 — Table S1: Overview of the augmentation factors and clinical histories of recreationally active and trained individuals with challenge‐confirmed WALDA. Table S2: Patient‐reported outcomes from recreationally active and trained individuals with WALDA: Responses to post‐challenge questionnaire (as described in 2). [file SMS-35-e70134-s001.docx]

**Supplementary material**

**Clinical characteristics and disease burden of wheat allergy dependent on augmentation factors (WALDA) in recreationally active and trained individuals**

Valentina Faihs, Claudia Kugler, Rebekka K. Linhart, Julia Felicitas Pilz, Tilo Biedermann, Knut Brockow

**Table S1.** Overview of the augmentation factors and clinical histories of recreationally active and trained individuals with challenge-confirmed WALDA.

| **Patient number** | **Gender** | **Age** | **Exercise session/week** | **Type of exercise performed** | **Tier according to ^1^** | **Exercise inducing reactions** | **Reactions during / after exercise** | **Identified exercise that never led to reactions?** | **Exercise intensity at reactions** | **Symptoms at worst reaction** | **Reduced / discontinued exercising due to reactions?** | **Single augmentation factors other than exercise** | **Secondary augmentation factors in exercise-induced reactions** |
| --- | --- | --- | --- | --- | --- | --- | --- | --- | --- | --- | --- | --- | --- |
| 1 | f | 29 | 4 | dancing, skiing, running, cycling | 2 | dancing, running | during | skiing | low-middle | urticaria, gastrointestinal symptoms, and needed CPR at 3 reactions | dicontinued | none | none |
| 2 | m | 36 | 6 | weight training, cross trainer, swimming, running | 1 | cross trainer, swimming, running | during | weight training | high | urticaria, diarrhea, vomiting, unconsciousness | reduced | NSAID | NSAID |
| 3 | m | 39 | 2 | running, gym | 1 | running, walking | during | weight training | low | urticaria | no | NSAID, tolperisone, stress | none |
| 4 | f | 55 | 4 | running | 1 | running | during |  | middle-high | urticaria, tightness of the throat, hypotension | reduced | NSAID | cold |
| 5 | f | 56 | 2 | running, cycling, hiking, tennis, cross-country skiing | 1 | walking, cycling | during | none | low | urticaria, unconsciousness | no | none | stress |
| 6 | m | 45 | 3 | swimming, weight training | 1 | walking | during | swimming, weight training | low | urticaria, unconsciousness, severe hypotension (blood pressure not measurable) | no | infections, vaccination, alcohol, sauna | stress, cold and heat |
| 7 | m | 39 | 2 | running, hiking | 1 | running | during | weight training | middle | urticaria, angioedema, dizziness | reduced | none | alcohol |
| 8 | f | 30 | 4 | running | 1 | running | after |  | high | urticaria, angioedema, dyspnea, vomiting and diarrhea, hypotension, unconsciousness | reduced | alcohol | heat, alcohol |
| 9 | m | 52 | 3 | cycling, weight training | 1 | cycling | during | weight training | middle-high | urticaria, throat tightness, hypotension | no | none | heat |
| 10 | m | 51 | 4 | running, hiking, cycling, skiing, weight training | 2 | running, hiking, skiing | during | weight training | high | urticaria, angioedema, tachycardia | no | none | alcohol, heat |
| 11 | m | 62 | 5 | walking, running, cycling | 1 | cycling | during | walking, running | high | urticaria | no | NSAID | NSAID and alcohol |
| 12 | m | 36 | 5 | running, hiking, weight training, martial arts | 2 | running, hiking | during | weight training, martial arts | low-high | urticaria, tachycardia | no | none | stress, alcohol, heat |
| 13 | m | 44 | 7 | running (marathons), cycling, skiing | 2 | running | during | cycling, skiing | high | urticaria, unconsciousness | no | NSAID | sleep deprivation |
| 14 | m | 57 | 2 | ski touring, hiking | 1 | ski touring, hiking | during |  | middle-high | urticaria | no | none | none |
| 15 | m | 33 | 2 | soccer | 2 | soccer | during |  | high | urticaria, dyspnea, tachycardia, hypotension, unconsciousness | reduced | none | stress, heat |
| 16 | m | 56 | 3 | ski mountaineering, tennis, mountainbiking, hiking | 1 | hiking, tennis | during | none | middle | urticaria, hypotension, unconsciousness | no | infection+NSAID+ stress | stress, heat |
| 17 | f | 23 | 7 | running, cycling, hiking, swimming, weight training | 1 | walking, dancing, hiking, swimming | during | weight training | low | urticaria, angioedema, nausea, dizziness | no | none | NSAID, stress, heat/cold |
| 18 | m | 45 | 2 | soccer | 1 | soccer | during | none | middle | urticaria, dyspnea, hypotension, presyncope | no | stress | heat |
| 19 | m | 53 | 3 | running, gym, cycling | 1 | walking | during | running, gym, cycling | low | urticaria, shortness of breath, unconsciousness | no | none | alcohol, heat, stress |
| 20 | m | 50 | 4 | running, cycling | 1 | running, soccer, walking home after eating and paddle tennis | during | cycling | low-high | urticaria, shortness of breath, unconsciousness | reduced - stopped runnning | none | alcohol, heat/cold |

Abbreviations: CPR, cardiopulmonary resuscitation; f, female; m, male; pat., patient; NSAID, non-steroidal anti-inflammatory drugs; WALDA, wheat allergy dependent on augmentation factors.1. McKay AKA, Stellingwerff T, Smith ES, et al. Defining training and performance caliber: A participant classifcation framework. *International Journal of Sports Physiology and Performance* 2022;**17**:317-31.

**Table S2.** Patient-reported outcomes from recreationally active and trained individuals with WALDA: Responses to post-challenge questionnaire (as described in ^2^).

| **1** | **2** | **3** | **4** | **5** | **6** | **7** | **8** | **9** | **10** | **Median points** | Percentage of selected responses | |
| --- | --- | --- | --- | --- | --- | --- | --- | --- | --- | --- | --- | --- |
| **1. How would you rate your QOL before you first suffered a severe allergic reaction in WALDA?**  (1 = extremely poor to 10 = exceptionally good) | | | | | | | | | | |  | <5% |
|  |  |  |  |  |  |  |  |  |  |  |  | 5.0-10% |
|  | | | | | | 5 % | 25 % | 50 % | 5 % | 9.0 |  | 11-20% |
| **2. How would you rate your QOL after suffering a severe allergic reaction in WALDA for the first time, but before being diagnosed?** (1 = extremely poor to 10 = exceptionally good) | | | | | | | | | | |  | 21-30% |
|  |  |  |  |  |  |  |  |  |  |  |  | >31% |
|  | 5 % | 5 % |  | 10 % | 20 % | 25 % | 10 % | 10 % |  | 7.0 |  |  |
| **3. How would you rate your QOL since you were informed and advised about the diagnosis of WALDA?**  (1 = extremely poor to 10 = exceptionally good) | | | | | | | | | | |  |  |
|  |  |  |  |  |  |  |  |  |  |  |  |  |
|  | | | | |  | 20 % | 25 % | 25 % | 5 % | 8.0 |  |  |
| **4. How strong would you rate your fear of having another allergic reaction after having suffered a severe allergic reaction in WALDA for the first time, but before being diagnosed?** (1 = no fear to 10 = strongest imaginable fear) | | | | | | | | | | |  |  |
|  |  |  |  |  |  |  |  |  |  |  |  |  |
| 10 % |  | 10 % | 5 % | 5 % | 5 % | 15 % | 20 % |  | 5 % | 7.0 |  |  |
| **5. How strong would you rate your fear of having another allergic reaction since you were informed and advised about the diagnosis of WALDA?** (1 = no fear to 10 = strongest imaginable fear) | | | | | | | | | | |  |  |
|  |  |  |  |  |  |  |  |  |  |  |  |  |
| 10 % | 25 % | 15 % | 5 % | 15 % |  |  |  | | | 3.0 |  |  |
| **6. How well-informed do you feel about WALDA and the current state of research?**  (1= I do not feel informed at all to 10= I feel very well informed) | | | | | | | | | | |  |  |
|  |  |  |  |  |  |  |  |  |  |  |  |  |
|  | | | 5 % |  |  | 5 % | 20 % | 20 % | 25 % | 9.0 |  |  |
| **7. How confident do you feel in dealing with your illness?** (1 = completely unconfident to 10 = absolutely confident) | | | | | | | | | | |  |  |
|  |  |  |  |  |  |  |  |  |  |  |  |  |
|  | | | | | 5 % | 10 % | 15 % | 30 % | 15 % | 9.0 |  |  |
| **8. How strong do you rate your initial fear of the completed OCT?** (1 = no fear to 10 = strongest imaginable fear) | | | | | | | | | | |  |  |
|  |  |  |  |  |  |  |  |  |  |  |  |  |
| 15 % | 30 % | 25 % | 5 % |  | | 5 % | 5 % |  |  | 2.0 |  |  |
| **9. How stressful did you find the OCT?** (1 = not stressful at all to 10 = extremely stressful) | | | | | | | | | | |  |  |
|  |  |  |  |  |  |  |  |  |  |  |  |  |
| 10 % | 30 % | 15 % | 15 % | 5 % |  |  |  |  |  | 2.0 |  |  |
| **10. How would you rate the benefit of the OCT for yourself?** (1 = extremely poor to 10 = extremely good) | | | | | | | | | | |  |  |
|  |  |  |  |  |  |  |  |  |  |  |  |  |
|  | | | | 5 % | 5 % | 5 % | 5 % | 10 % | 45 % | 10.0 |  |  |

Coloured values in columns 1-10 represent the percentage of patients selecting each response option. The colour legend on the right indicates the colour coding for different percentage ranges.

Missing values: n=3 to 5 of 20 included recreational athletes with challenge-confirmed WALDA.

Abbreviations: OCT, oral challenge test; QOL, quality of life; WALDA, wheat allergy dependent on augmentation factors.

2 .Faihs V, Kugler C, Bent R, Biedermann T, Brockow K. Challenge-confirmed diagnosis restores quality of life in cofactor-dependent wheat allergy. *Annals of Allergy, Asthma &*Immunology2023;131:494–500.e1.
